# Doped zirconia phase and luminescence dependence on the nature of charge compensation

**DOI:** 10.1038/srep44453

**Published:** 2017-03-13

**Authors:** Krisjanis Smits, Dags Olsteins, Aleksejs Zolotarjovs, Katrina Laganovska, Donats Millers, Reinis Ignatans, Janis Grabis

**Affiliations:** 1 Institute of Solid State Physics, University of Latvia, Riga, Latvia; 2 Institute of Inorganic Chemistry, Riga Technical University, Latvia

## Abstract

Zirconia is a relatively new material with many promising practical applications in medical imaging, biolabeling, sensors, and other fields. In this study we have investigated lanthanide and niobium doped zirconia by luminescence and XRD methods. It was proven that charge compensation in different zirconia phases determines the incorporation of intrinsic defects and activators. Thus, the structure of zirconia does not affect the Er luminescence directly; however, it strongly affects the defect distribution around lanthanide ions and the way in which activator ions are incorporated in the lattice. Our results demonstrate the correlation between the crystalline phase of zirconia and charge compensation, as well as the contribution of different nanocrystal grain sizes. In addition, our experimental results verify the theoretical studies of metastable (tetragonal, cubic) phase stabilization determined using only oxygen vacancies. Moreover, it was found that adding niobium drastically increases activator luminescence intensity, which makes Ln^3+^ doped zirconia even more attractive for various practical applications. Although this study was based on the luminescence of the Er ion, the phase stabilization, charge compensation, and luminescence properties described in our results are expected to be similar for other lanthanide elements. Our results suggest that the luminescence intensity of other oxide matrices where lanthanides incorporate in place of tetravalent cations could be increased by addition of Nb ions.

Zirconia (ZrO_2_) is considered to be an excellent material for applications in optics because of its wide bandgap, good transparency, high refractive index, and high material hardness. The phonon energy of ZrO_2_ is low; therefore, its thermal quenching luminescence is less efficient than in most other oxide materials.

Zirconia ceramics are already widely used in biological implants. Therefore zirconia nanocrystals are expected to be non-toxic. The luminescent characteristics of Er^3+^ doped zirconia have various possible applications and have attracted strong scientific interest in the fields of biology and medicine. However, the main disadvantage of zirconia is its relatively weak luminescence compared to fluorides or oxysulfides. Recently, we have demonstrated that intrinsic defects in zirconia are responsible for a reduction in Er^3+^ upconversion luminescence intensity^1^, and that this luminescence intensity reduction can be avoided by adding Nb^5+ ^2; however, the mechanisms behind this effect were not clear, and additional study was required. A reanalysis of these results suggests that the structure of zirconia and the stabilization processes provide the keys to increasing the luminescence intensity. At room temperature and ambient pressure, zirconia usually exists in monoclinic phase; however, the tetragonal and cubic phases can be stabilized by adding dopants with lower valence^3^ or by increasing the surface energy in the case of nanocrystals (nanocrystal sizes below 30 nm)^4^. Oxygen vacancies play a major role in the stabilization of tetragonal or cubic phases in zirconia. Phase stabilization using only oxygen vacancies has been confirmed by theoretical calculations^5^. In addition to the phase stabilization, oxygen vacancies also strongly affect the Ln^3+^ luminescence intensity and spectral distribution^1^,^6^,^7^. Two effects were observed when increasing the Ln^3+^ ion content: the luminescence intensity in zirconia is concentration-dependent, and zirconia phases change from monoclinic to tetragonal or even to cubic phase. For tetragonal phase stabilization, the addition of up to 6% of trivalent dopants usually is required, whereas the required concentration for achieving the cubic phase is 10–12%^8^.

In the case of a low Ln^3+^ concentration, the size of the crystallites leads to an initial phase that can be tetragonal or monoclinic, depending on the method of synthesis. Higher annealing temperatures lead to larger grain size, thus reducing the surface defect impact on the nonradiative recombination of excitations. Both the defects (in volume or surface defects) and structure affect the luminescence intensity. This complex situation makes it difficult to compare results obtained by different researchers and complicates the search for optimal parameters for obtaining the highest possible Ln^3+^ ion luminescence intensity.

The impact of zirconia structure on Ln^3+^ luminescence is still not completely determined, and the optimal concentrations of Ln^3+^ and Nb are not clear. Therefore, the present study is focused on how the phase impacts on the luminescence and on the selection of the optimal Ln^3+^ and Nb ion concentrations for achieving maximum luminescence intensity. Since it is a second order process and thus more sensitive to changes in local symmetry, mainly upconversion luminescence was used instead of downconversion luminescence.

## Results and Discussions

Our previous research has shown that Nb ions incorporated in the lattice strongly increase the Er luminescence intensity, especially the upconversion processes. The intensity increase was explained by the charge compensation, where the optimal Nb^5+^ content was the same as the Ln^3+^ content. Furthermore, as a result of adding Nb, the Ln^3+^ ions are expected not to take part in the tetragonal phase stabilization process, and thus the formation of the monoclinic phase should be dominant. As the structure of zirconia also depends on crystallite size, the samples were also annealed at different temperatures.

**Figure 1:**
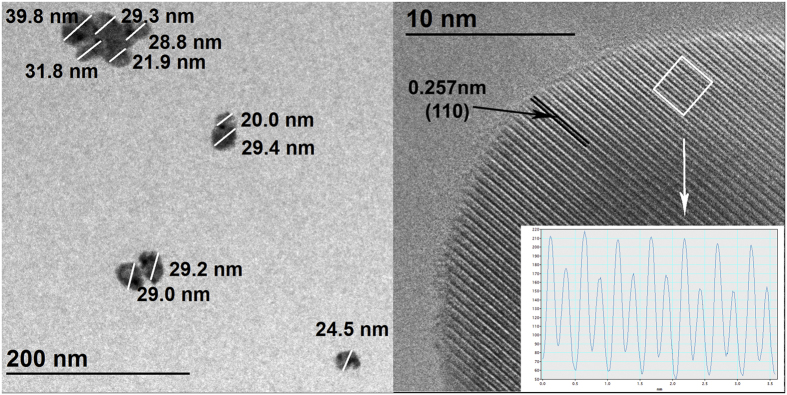
TEM (left) and HrTEM (right) images of sample 2Er annealed at 800 °C.

For samples annealed at 800 °C the nanocrystal grain sizes were in the 15–30 nm range, and the tetragonal phase was dominant. Transmission electron microscope (TEM) images showed that samples annealed at the lowest temperature (800 °C) already have a good crystal structure (Fig. 1).

**Figure 2:**
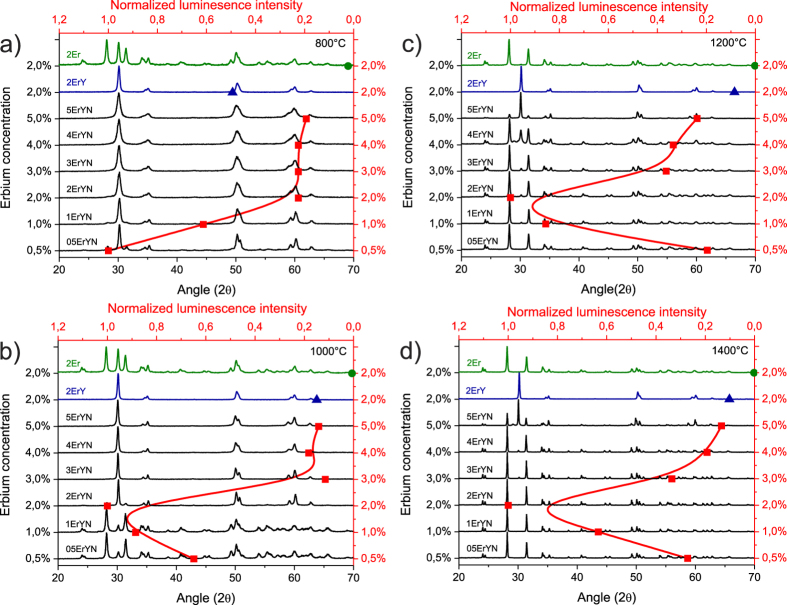
The zirconia structure dependence on Er, Yb, and Nb ion content and annealing temperatures (XRD) with the upconversion luminescence intensities plotted for each sample.

Phase transformation with increasing annealing temperature can be seen in the X-ray diffraction (XRD) data for all samples with Nb (Fig. 2). The nanocrystal grain sizes become larger, and the excess surface energy is not sufficient to stabilize the tetragonal structure. In addition, even large quantities of Ln^3+^ ions do not stabilize the tetragonal phase as a result of additional doping of Nb; therefore, the phase transition from tetragonal to monoclinic phase occurs (Fig. 2d).

Despite the high Er and Yb concentrations, only the sample 5ErYN with a high concentration of dopants (5% Er, 10% Yb, 15% Nb) remained in the tetragonal phase and contained some amount of niobates. This result can be explained by the large percentage of dopants (30%), which caused them to form aggregates and resulted in new niobate or niobium-zirconate phases.

For samples annealed at 800 °C the most intense upconversion luminescence was observed in sample 05ErYN (0.5% Er, 1% Yb, 1.5% Nb) with the lowest Er content; when the Er concentration was increased, the upconversion luminescence intensity decreased. This observation correlates with the results obtained for Er doped zirconia^9^ and also for Er and Yb doped zirconia, where the optimal concentration of Er and Yb was found to be 0.5 at % Er and 2 at % Yb^10^,^11^.

For samples containing Nb ions and annealed at more than 800 °C, the luminescence dependence on Er content changes drastically: upconversion luminescence intensity increases as the Er concentration is increased up to 2%.

The sample 2ErYN (with 2% Er, 4% Yb, and 6% Nb) annealed at 1400 °C showed the most intense luminescence. This observation correlates with other materials where the most intense green upconversion luminescence was also found for samples with concentrations of Er ions around 2%^12^,^13^.

**Figure 3:**
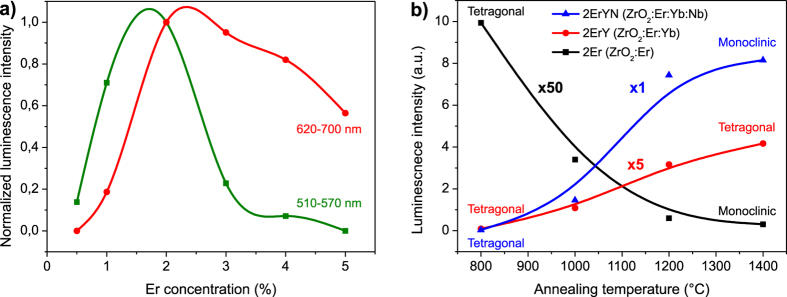
Integral luminescence dependence on Er concentration for Nb doped samples (**a**) and upconversion luminescence intensities for samples 2Er, 2ErY, and 2ErYN annealed at different temperatures (**b**).

The increase of the intensity of the red (^4^F_9/2_ → ^4^I_15/2_) Er ion luminescence band for heavily doped samples does not compensate the loss of the green band (^4^S_3/2_ → ^4^I_15/2_) (Fig. 3a); however, by changing the Er ion concentration, the ratio between the red and green luminescence bands can be tuned, which can be used to make the sample more suitable for a particular application.

For samples with Nb one might assume that the most efficient upconversion luminescence can be attributed to the monoclinic zirconia structure; however, that is not the case: other researchers ascribed the most intense Er upconversion luminescence to the tetragonal zirconia phase^1^,^11^,^14^,^15^. Our previous research indicates that the luminescence intensity and spectral distribution is dependent more on structure related defects and less on the local crystal field differences between monoclinic and tetragonal structures^1^.

To show the impact of dopants on the zirconia phase, we analysed two sets of samples without Nb: one doped only with Er and the other with Er and Yb, thus allowing us to compare three different samples (zirconia phases) with the same Er content. For the sample 2Er (with 2%Er ion doping) a tetragonal to monoclinic phase transfer was observed as the grain size (annealing temperature) was increased; however, at the same time the upconversion luminescence intensity rapidly decreased (Fig. 3b). In sample 2ErY (2%Er and 4%Yb) the total Ln^3+^ ion concentration is 6%, which is sufficient for tetragonal phase stabilization. The luminescence of this sample shows an increase of intensity with an increase of the annealing temperature. This correlation can be explained by a reduction of the impact of surface defects as the grain size decreases. In addition, the distribution of Ln^3+^ ions became more homogeneous. To sum up, the samples 2Er and 2ErYN had the same phase transformation behaviour (tetragonal to monoclinic), but opposite luminescence intensity dependence (Fig. 3b). This statement clearly shows that the upconversion luminescence intensity dependence cannot be explained by local crystal fields in different structures, as was attempted in previous studies. Therefore, question of why the structural changes have such a strong impact on upconversion luminescence of Er ions remains unanswered. The answer could be related to how the Ln^3+^ ions are incorporated in a particular crystal structure and are affected by intrinsic defects.

For sample 2Er at low annealing temperature, the tetragonal phase was stabilized by surface energy and oxygen vacancies, leading to a high probability of homogeneous (uniform) Er distribution. There is no need for oxygen vacancies or homogeneous vacancy distribution in monoclinic zirconia. Thus, when the tetragonal to monoclinic phase transfer occurs, the oxygen vacancy concentration decreases and the remaining oxygen vacancies settle close to Er ions. In addition, with an increase of annealing temperature, the probability of Er diffusion increases, and the Er ions can agglomerate. It should be noted that two Er ions are required for charge compensation of a single oxygen vacancy. We suggest that with an increase of annealing temperature, the oxygen vacancies relocate between two Er ions or the Er ions relocate closer to each other.

**Figure 4:**
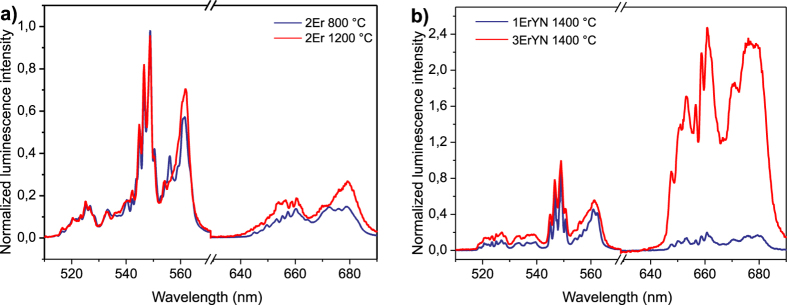
Upconversion luminescence spectra for sample 2Er annealed at 800 °C and 1400 °C (**a**) and for samples 1ErYN and 3ErYN (**b**).

The green luminescence spectral distribution of sample 2Er annealed at 800 °C and 1400 °C was almost the same (Fig. 4a). This means that, despite phase transformations, the surroundings of Er ions do not change significantly. At the same time, the strong decrease in luminescence intensity means that the amount of Er ions participating in the luminescence is strongly reduced. The defect relocalization or Er relocalization completely quenches these Er luminescence centres. The red luminescence slightly increases with the increase of annealing temperature, indicating that the mutual separation of Er ions becomes smaller (Fig. 4a), thus increasing the probability of cross relaxation processes. A similar effect could be observed for the samples with Nb, when the Er concentration was increased (Fig. 4b). However, with the increase of Er content, the “red” luminescence part increased drastically. Therefore it is not possible to explain luminescence quenching by Er ion aggregation for sample 2Er. In addition, the cation diffusion in tetragonal Ce and Yb doped zirconia was noticed in samples annealed at 1200 ° C, whereas anion diffusion is six orders of magnitude faster and occurs at much lower temperatures^16^. Taking these facts into account, one can conclude that the strong luminescence quenching for 2Er sample when annealed at over 800 °C is related to phase transitions and diffusion in the oxygen sublattice. Er^3+^ incorporates in Zr^4+^ sites, which leads to higher D4d symmetry in the tetragonal phase (4/mmm symmetry) and lower C2h symmetry in the monoclinic phase (2/m symmetry). Lowering the site symmetry of the Er ion will cause an increase in transition probabilities of Er^3+^ ions, and in consequence a higher luminescence for Er^3+^ ions incorporated in the monoclinic phase^14^. However, the opposite effect is observed, which indicates that the luminescence quenching can be explained only by intrinsic defects.

**Figure 5:**
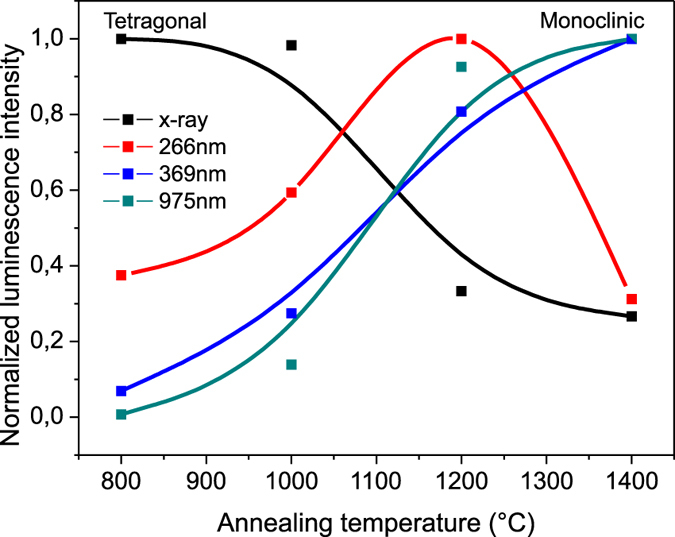
Dependence of differently excited luminescence on the annealing temperature for sample 2ErYN.

By changing the excitation source, we studied how the Er ions are excited. This experiment was performed for the sample with the highest luminescence intensity 2ErYN annealed at different temperatures (Fig. 5). The dependence of the photoluminescence from direct Er ion excitation (^4^I_15/2_ → ^2^G_7/2,_^4^G_7/2,_^2^K_15/2_) on annealing temperature was very similar to the upconversion luminescence results. Therefore, the phase and defect impact is not related to particular upconversion phenomena, but rather to the Er ion photoluminescence as a whole.

The results for luminescence excited by X-rays and by 266 nm radiation differed. In both cases the energy transfer from intrinsic zirconia defects to the Er ion is responsible for luminescence. With the increase of annealing temperature, oxygen vacancy and surface defect concentration reduces, thereby also reducing the efficiency of energy transfer from intrinsic defects to Er ions, and thus decreasing the luminescence intensity.

**Figure 6:**
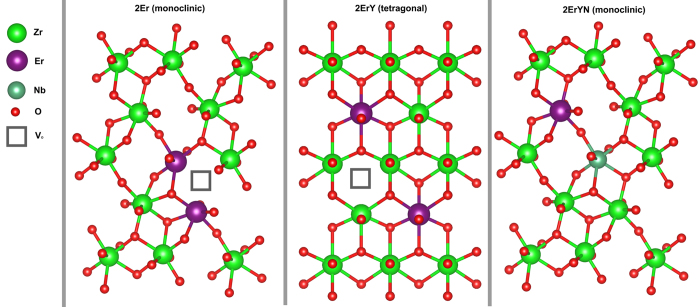
Possible mechanisms for the incorporation of Er ion and oxygen vacancies in tetragonal, monoclinic and Nb doped monoclinic zirconia.

The Extended X-Ray Absorption Fine Structure (EXAFS) spectra analysis showed that in the tetragonal and cubic phases mostly oxygen vacancies rather than dopants are associated with the Zr^17^,^18^. Unfortunately, we could not find EXAFS data on trivalent activators in the monoclinic zirconia phase in the literature. It is possible that in the monoclinic phase, the oxygen vacancies relocate closer to Er ions. In the case of samples with Nb, the addition of Nb in the same quantity as the trivalent activators reduces the number of oxygen vacancies^19^,^20^. An illustration of possible Er incorporation mechanisms in monoclinic and tetragonal phases is shown in Fig. 6.

The concentration quenching of the ZrO_2_:Er:Yb:Nb system is mainly related to Er ion concentration. Potentially, the Yb content could be increased further, leading to higher absorption efficiency, and thus a more efficient emission^21^.

Similar charge compensations effect is expected to appear in other oxide matrices; especially 4th group transition metal oxides. For example hafnia which is isostructural to zirconia and with similar luminescence properties^22^ as well as titania which is one of the famous materials for photocatalysis in which the upconversion process could be applied as well^23^,^24^.

## Summary

The experimental results of our study correlate well with theoretical calculations in the literature^5^. Oxygen vacancies are the main agents in the phase stabilization in zirconia.

The main reason for the change in luminescence intensity with zirconia phase transfer is not associated with the difference in crystal symmetry, but rather with the relocalization of Er ions and intrinsic defects.

We have demonstrated the impact of Nb doping on the structural and luminescence properties of zirconia. By using Nb doping the luminescence intensity of the Er ion (and possibly other rare earth ions) can be significantly increased, thus making this material even more interesting for practical applications. When adding Nb, the optimal Er ion concentration for most intense luminescence is about 2%, which is similar to results obtained for fluoride materials.

The concentration quenching of the ZrO_2_:Er:Yb:Nb system and other materials is mainly related to Er ion concentration. The Yb content could potentially be increased further, leading to higher absorption efficiency, and thus more efficient emission. It is expected that similar phenomena will occur for all Ln^3+^ activators in zirconia. Our results suggest the need to review studies of materials in which lanthanides incorporate in place of tetravalent cations.

## Methods

We chose Er ions as the dopants to produce luminescence centres in samples; additional doping with Yb increases the absorption cross-section, thus improving the overall efficiency of the upconversion luminescence process. The Nb ions will be added to compensate the charge differences caused by adding Er and Yb ions.

Samples of zirconia doped with Er, Yb, and Nb were prepared by the Sol-Gel method. Samples without Nb were also prepared for comparison in the context of our previous research that described the role of Nb in phase stabilization and luminescence increase^2^.

By taking into account our previous research results and to reduce the amount of necessary samples we fixed the Er, Yb and Nb ratio to 1:2:3, with the following Er ion concentrations: 0.5, 1, 2, 3, 4, and 5 at %. Additional samples with only Er doping (2%) and Er, Yb doping (2% Er and 4% Yb) were prepared to clear up the effect of Nb and annealing on the structure and luminescence properties. All samples described in this paper are listed in Table 1. The Sol-Gel sample synthesis method has been described previously^1^,^2^. After synthesis, each sample was split into four parts and annealed at 800, 1000, 1200, and 1400  °C for two hours. The results obtained from a study of 32 samples are presented.

To check the final dopant content and detect unexpected impurities, an energy dispersive X-ray analysis (EDX) of the samples using the S4 Pioneer (Bruker AXS) was performed. The crystalline structure of the samples was examined via X-ray diffraction (XRD) using an X-ray diffractometer (X’Pert Pro MPD) with Cu-Kα radiation (λ = 0.154 nm). The crystalline size verification and morphology studies were performed using a transmission electron microscope (TEM, Tecnai G2 F20, FEI) operated at 200 kV. The samples for TEM studies were placed on a holey carbon coated grid AGS147-4 (Agar Scientific)^21^.

The luminescence measurements were carried out at room temperature using four different excitation sources: a 975-nm Thorlabs L975P1WJ laser diode coupled with a Thorlabs ITC4005 controller, an LED with an emission maximum at 376 nm, a YAG laser FQSS266 (CryLas GmbH) 4th harmonic at 266 nm (4.66 eV), and an X-ray tube with a tungsten anode operated at 30 kV and 10 mA.

The samples for luminescence measurements were pressed into equally sized stainless steel holders, so that the geometries of the excitation-registration channels were mostly identical, and comparison of the luminescence intensities of the samples was possible.

The luminescence spectra were recorded using an Andor Shamrock B-303i spectrograph equipped with a CCD camera (Andor DU-401A-BV) and Horiba iHR320 spectrograph coupled with a photomultiplier tube (Hamamatsu R928P).

## Additional Information

**How to cite this article**: Smits, K. *et al*. Doped zirconia phase and luminescence dependence on the nature of charge compensation. *Sci. Rep.* **7**, 44453; doi: 10.1038/srep44453 (2017).

**Publisher's note:** Springer Nature remains neutral with regard to jurisdictional claims in published maps and institutional affiliations.

## Figures and Tables

**Table 1:** List of samples.

Sample	Dopants (at %)			Annealing temperature (°C)							
	Er	Yb	Nb	800		1000		1200		1400	
				Phase (%)	Size (nm)	Phase (%)	Size (nm)	Phase (%)	Size (nm)	Phase (%)	Size (nm)
2Er	2	—	—	M:70 T:30	M:32 T:33	M:70 T:30	M:45 T:43	M:97 T:3	M:88 T:-	M:100 T:0	M:143 T:-
2ErY	2	4	—	M:0 T:100	M:- T:30	M:0 T:100	M:- T:46	M:0 T:100	M:- T:101	M:0 T:100	M:- T:162
05ErYN	0.5	1	1.5	M:28 T:72	M:19 T:27	M:85 T:15	M:32 T:-	M:100 T:0	M:57 T:-	M:100 T:0	M:94 T:-
1ErYN	1	2	3	M:13 T:87	M:- T:21	M:86 T:14	M:27 T:-	M:97 T:3	M:72 T:-	M:100 T:0	M:100 T:-
2ErYN	2	4	6	M:0 T:100	M:- T:15	M:15 T:85	M:- T:39	M:97 T:3	M:60 T:-	M:100 T:0	M:112 T:-
3ErYN	3	6	9	M:0 T:100	M:- T:16	M:0 T:100	M:- T:42	M:95 T:5	M:66 T:-	M:98 T:2	M:113 T:-
4ErYN	4	8	12	M:0 T:100	M:- T:15	M:0 T:100	M:- T:30	M:27 T:72	M:56 T:63	M:96 T:4	M:112 T:-
5ErYN	5	10	15	M:0 T:100	M:- T:15	M:0 T:100	M:- T:35	M:9 T:91	M:- T:60	M:42 T:58	M:109 T:94

As the Yb and Nb contents are proportional to Er content, only Er content is included in sample names. The letters M and T refer to monoclinic and tetragonal phase.
